# COVID-19 Rapid Antigen Test Screening in Patients on Hemodialysis

**DOI:** 10.1155/2022/4678717

**Published:** 2022-09-14

**Authors:** Gaetano Alfano, Roberta Scarmignan, Niccolò Morisi, Francesco Fontana, Silvia Giovanella, Giulia Ligabue, Laura Rofrano, William Gennari, Monica Pecorari, Mariacristina Gregorini, Gianni Cappelli, Riccardo Magistroni, Gabriele Donati

**Affiliations:** ^1^Nephrology, Dialysis and Transplant Unit, University Hospital of Modena, Modena, Italy; ^2^Surgical, Medical and Dental Department of Morphological Sciences, Section of Nephrology, University of Modena and Reggio Emilia, Via Del Pozzo 71, Modena 41124, Italy; ^3^Clinical and Experimental Medicine Program, University of Modena and Reggio Emilia, Via Del Pozzo 71, Modena 41124, Italy; ^4^Clinical Microbiology Unit, University Hospital of Modena, Modena, Italy; ^5^Molecular Microbiology and Virology Unit, Department of Laboratory Medicine and Pathological Anatomy, Azienda Ospedaliero Universitaria di Modena, Modena, Italy; ^6^Nephrology and Dialysis, AUSL-IRCCS Reggio Emilia, Reggio Emilia, Italy

## Abstract

**Introduction:**

Patients receiving in-center hemodialysis are extremely vulnerable to COVID-19. It is unclear if routine screening of asymptomatic hemodialysis patients is an effective strategy to prevent COVID-19 outbreaks within the dialysis unit.

**Methods:**

We conducted a retrospective analysis of in-center hemodialysis patients who underwent bimonthly COVID-19 rapid antigen test screening from February 15^th^ to December 26^th^, 2021. Nasal rapid antigen testing was performed in all asymptomatic patients. All rapid antigen-positive tests were confirmed by RT-PCR nasopharyngeal swab. Besides universal rapid antigen screening, RT-PCR testing was conducted in all symptomatic patients and contacts of COVID-19 subjects.

**Results:**

Overall, 4079 rapid antigen tests were performed in 277 hemodialysis patients on chronic hemodialysis with a mean age of 68.4 ± 14.6 years. Thirty-eight (0.9%) rapid antigen tests resulted positive. Only five (13.8%) positive-rapid antigen tests were also positive by RT-PCR testing. During the same period, 219 patients regularly screened by rapid antigen tests bimonthly underwent 442 RT-PCR nasopharyngeal swabs for clinical reasons. RT-PCR testing yielded a positive result in 13 (5.9%) patients. The time elapsed between PCR and the negative-rapid antigen test was 7.7 ± 4.6 days (range 1.8–13.9 days). At the end of the follow-up, 6.4% of the population on in-center hemodialysis contracted COVID-19, and routine rapid antigen tests detected only 5 out of 18 (27.7%) COVID-19 cases. No outbreaks of COVID-19 were identified within the dialysis unit.

**Conclusion:**

Bimonthly rapid antigen screening led to the early diagnosis of COVID-19 in less than one-third of cases. The short incubation period of the new SARS-CoV-2 variants makes bimonthly test screening inadequate for an early diagnosis of COVID-19. More frequent tests are probably necessary to improve the utility of COVID-19 nasal rapid antigen test in patients on hemodialysis.

## 1. Introduction

Patients on maintenance hemodialysis are extremely susceptible to the consequences of COVID-19 [1]. The most common determinants of poor outcomes are advanced age and the burden of comorbidities, including end-stage kidney disease [2]. Patients receiving in-center hemodialysis also carry a significant risk of contracting SARS-CoV-2 infection because they share enclosed spaces including public transportation, dressing rooms, and dialysis rooms. For these reasons, routine screening of hemodialysis patients for COVID-19 has been proposed as an ideal solution for the early detection of asymptomatic cases and to prevent deadly outbreaks within the dialysis unit [3]. To date, the experience of the universal screening of hemodialysis patients is limited to the use of molecular COVID-19 testing along with the radiologic assessment of the lungs [1, 4]. The performance of rapid antigen test has not yet been verified in patients on hemodialysis. This study aimed to describe the experience of routine rapid antigen screening in asymptomatic patients undergoing in-center maintenance hemodialysis.

## 2. Methods

The electronic charts of patients on chronic hemodialysis treatment from February 15th to November 15th, 2021, were reviewed retrospectively. Rapid antigen test screening was performed every two weeks as a universal screening for asymptomatic patients. Rapid antigen testing was performed on the anterior nasal swab and processed using the LIAISON® SARS-CoV-2 Ag assay, a chemiluminescence sandwich-immunoassay (CLIA) based technology for the quantitative detection of SARS-CoV-2 antigens (nucleocapsid antigen protein). Manufacturer's information reported clinical sensitivity and specificity on nasal samples positive for real-time (RT)-PCR within 10 days of the onset of symptoms of 98.0% and 99.5%, respectively. According to our protocol, body temperature, masking, COVID-19 symptoms, and handwashing were checked before dialysis room entry. All health workers of our hospital received two doses of the mRNA COVID-19 vaccine and were screened every three weeks with nasopharyngeal swab PCR testing. The rapid antigen test was performed as a universal screening in asymptomatic patients through a nasal swab by trained nurses during the dialysis treatment. All patients with an antigen-positive test underwent a confirmatory nasopharyngeal swab RT-PCR testing within a few hours of the antigen test. Two RT-PCR assays were used for the qualitative detection of nucleic acid from SARS-CoV-2 in respiratory specimens: the Alinity *m* System SARS-CoV-2 kit (Abbott Molecular, Inc, Des Plaines, USA) and the Allplex SARS-CoV-2 Assay (Seegene, Seoul, Korea). The Alinity *m* SARS-CoV-2 assay detects N and RdRP genes of SARS-CoV-2 whereas Allplex SARS-CoV-2 Assay detects N, S and RdRP genes of SARS-CoV-2.

During the observational period, all patients screened by rapid antigen test underwent nasopharyngeal swab RT-PCR testing (symptom-based screening) in the case of COVID-19 symptoms (fever, cough, diarrhea, loss of smell and taste, nasal congestion and rhinorrhea, and vomit). RT-PCR testing was also performed in a minority of infected patients to end the isolation, screen contacts of COVID-19 subjects, and screen patients prior to surgical interventions.

Patients with positive RT-PCR results were placed in an isolated room until the resolution of the infection. Rapid antigen testing was performed neither to screen symptomatic patients nor to monitor viral shedding in COVID-19 patients.

A cost analysis was performed for rapid antigen and molecular testing. It included the cost of the containers, swab, and sample processions. The cost of the personnel involved in sample collection, transportation, and processing was not considered in the analysis. The average price of a single rapid antigen and RT-PCR test was estimated to be $5.4 and $19.6, respectively.

### 2.1. Statistical Analysis and Ethical Committee Approval

Baseline characteristics were described using the mean and standard deviation (SD). The percentage was used to describe categorical variables. All statistical analyses were performed using the SPSS 24® statistical software.

This study has been authorized by the local Ethical Committee of Emilia Romagna (n. 839/2020).

## 3. Results

**Figure 1:**
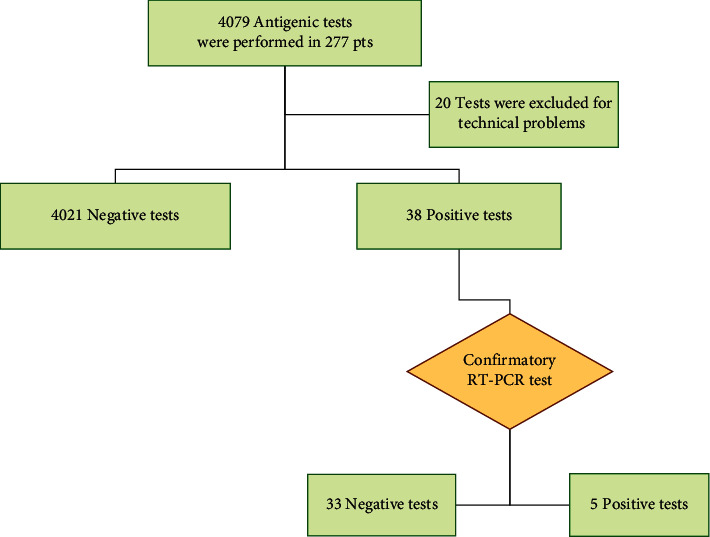
Flowchart of rapid antigen screening in patients on in-center hemodialysis.

Over 9 months, 4079 rapid antigen tests were performed in 277 asymptomatic patients on chronic hemodialysis (Figure 1). During this period of study, each patient received 14.7 rapid antigen tests as screening for COVID-19. The mean age of patients who underwent screening was 68.4 ± 14.6 years with a predominance of males (59.5%). Thirty-eight rapid antigen tests resulted in positive and only five (13.1%) were confirmed by RT-PCR testing (true positive) after 4.08 ± 4 hours from the rapid antigen test (Table 1). Three of these patients, asymptomatic at diagnosis, developed COVID-19-related symptoms a few days after the diagnosis of COVID-19.

During the same period, 219 patients underwent 442 RT-PCR nasopharyngeal swabs for clinical reasons (COVID-19 symptoms, end of isolation, etc.). Thirteen (24.3%) symptomatic patients resulted in positive for COVID-19. The time elapsed between the RT-PCR swab and the negative-rapid antigen test was 7.7 ± 4.6 days (range 1.8–13.9 days).

Overall, 18 out of 277 (6.4%) hemodialysis patients contracted COVID-19 during the study period. The mean age of these patients was 65.5 ± 16.1 years. Eight (44.4%) of them, including two vaccinated patients, died of severe complications of COVID-19 (Table 1).

A cost analysis of the diagnostic tests performed in the period under study revealed that the total cost of rapid antigen test screening accounted for $22 020.

## 4. Discussion

COVID-19 transmission within the hemodialysis unit should be firmly avoided as this illness is associated with mortality and morbidity among frail patients receiving hemodialysis. In this study, we evaluated the role of rapid antigen testing as a preventive measure for the spread of COVID-19. The rapid antigen test was indeed used for the screening of SARS-CoV-2 infection among asymptomatic in-center hemodialysis patients. We documented that bimonthly rapid antigen screening was able to detect less than one-third of all infected patients with COVID-19. To note, routine rapid antigen screening was also unable to detect COVID-19 during the incubation period of the illness in a small proportion of patients who became symptomatic a few days after the screening.

The rapid antigen test is now largely used for the diagnosis of SARS-CoV-2 infection and has been advocated as a preventive measure to detect COVID-19 in high-risk populations. However, the performance of this test is unclear in real-world clinical practice. The resulting sensibility of the rapid antigen test is extremely heterogeneous and tended to be lower than the cutoff of 80% recommended by the World Health Organization [5]. Recent findings showed a very low sensitivity (28.6%–77.8%) of rapid antigen tests among asymptomatic people [6–8].

The question that arises from this background is about the best strategy to adopt for the early detection of SARS-CoV-2 infection among hemodialysis patients. We think that the utility of the rapid antigen test cannot be overlooked in hemodialysis patients since it may be helpful for a quick diagnosis of COVID-19 in patients with new-onset symptoms requiring urgent hemodialysis treatment. Furthermore, rapid antigen testing might be a solution when RT-PCR is unavailable, the molecular lab is overwhelmed by the workload, or a prolonged turnaround time of RT-PCR analysis precludes its clinical utility [9, 10].

Based on our results, rapid antigen test screening has contributed, along with other measures, to mitigate the risk of the infection spreading within a high-risk congregate setting such as the hemodialysis population and led to the diagnosis and subsequent isolation of two COVID-19 patients who remained asymptomatic during the entire follow-up. However, the poor test performance in asymptomatic patients was insufficient to detect all cases of SARS-CoV-2 infection as COVID-19 incubation was generally shorter than 2-week screening interval. In particular, two SARS-CoV-2 strains spread in Italy during the study period between February and November 2021: the Alpha and Delta variants. By mid-February 2021, the ancestral SARS-CoV-2 strain was replaced by the Alpha variant which remained dominant in Italy until July 2021. In the second half of 2021, Delta variant became dominant in our country [11]. Evidence shows that Alpha and Delta variants had an estimated median incubation period of 4.5 [12] and 3.7 days [13, 14], respectively. Based on these data, we suppose that an increase in the test frequency (e.g., once a week) is probably an acceptable program to increase the sensibility of the diagnostic test. However, the economic impact of rapid antigen-based screening should be taken into account given the high health care expenditure due to the pandemic. In this context, the proposal of routine screening with RT-PCR screening is demanding as it has a much more relevant impact on economic health resources than rapid antigen test screening.

Given the lack of clear indications for the modality and timing of screening of these patients and the continued circulation of SARS-CoV-2, further efforts should be made to maintain symptom-based screening through RT-PCR and promote patient education on SARS-CoV-2 prevention. In particular, this group of patients must be alerted of their vulnerability to COVID-19 and discouraged from lifting restrictions within and outside of the dialysis unit.

The main limitations of this study are related principally to the performance of the rapid antigen test. As aforementioned, the short incubation period of SARS-CoV-2 and the low sensitivity of the rapid antigen test might have reduced the accuracy of the screening test. In addition, rapid antigen testing is expected to be less accurate in asymptomatic people than in people with signs or symptoms of infection. A recent Cochrane review confirmed that the rate of false-positive results in asymptomatic patients who underwent testing for COVID-19 was substantial (48%) [6]. Different sensitivity between diagnostic (genetic material vs. viral antigen) and sampling methods (nasal *vs*. nasopharyngeal swab) may explain the lower sensitivity of rapid antigen test compared to RT-PCR assay in detecting COVID-19 cases. Lastly, the large variability between tests produced by different manufacturers may affect the reproducibility and generalization of our results.

## 5. Conclusion

Although the rapid antigen test is more rapid and less expensive than RT-PCR test, bimonthly screening regimen was insufficient for assigning an early COVID-19 diagnosis in patients on hemodialysis. Further data about the performance of the rapid antigen testing in asymptomatic hemodialysis patients are required to establish the effective value of this test in limiting COVID-19 spread within the hemodialysis unit.

## Figures and Tables

**Table 1:** Demographic and clinical characteristics of all hemodialysis patients who contracted COVID-19.

Patient	Age (yr)	Sex	Test used for COVID-19 diagnosis	Time elapsed between antigenic and PCR tests (day)	Previous COVID-19 vaccination	Symptoms at COVID-19 diagnosis	Symptoms during the infectious period	Outcome
#1	80.5	F	Antigen test	—	No	No	Yes	Deceased
#2	74.4	F	Antigen test	—	No	No	No	Survived
#3	82.0	F	PCR	1.92	No	Yes	Yes	Deceased
#4	60.2	F	PCR	13.3	No	Yes	Yes	Survived
#5	89.5	M	PCR	11.9	No	Yes	Yes	Deceased
#6	68.5	F	PCR	7.7	No	Yes	Yes	Deceased
#7	81.6	M	Antigen test	—	No	No	Yes	Deceased
#8	74.4	M	PCR	1.89	No	Yes	Yes	Deceased
#9	46.6	M	Antigen test	—	No	No	Yes	Survived
#10	34.7	M	PCR	8.3	Yes	No	No	Survived
#11	51.9	M	Antigen test	—	Yes	No	No	Survived
#12	91.6	F	PCR	13.9	Yes	Yes	Yes	Deceased
#13	53.9	F	PCR	11	Yes	Yes	Yes	Survived
#14	53.7	F	PCR	3.4	Yes	Yes	Yes	Survived
#15	49.0	M	PCR	13.9	Yes	Yes	Yes	Survived
#16	59.0	M	PCR	2.4	Yes	Yes	Yes	Survived
#17	72.6	M	PCR	7.2	Yes	Yes	Yes	Deceased
#18	55.0	M	PCR	4.2	No	Yes	Yes	Survived

## Data Availability

All data included in this study are available upon reasonable request to the author.
